# Association Mapping of *Lathyrus sativus* Disease Response to *Uromyces pisi* Reveals Novel Loci Underlying Partial Resistance

**DOI:** 10.3389/fpls.2022.842545

**Published:** 2022-03-24

**Authors:** Davide Coelho Martins, Diego Rubiales, Maria Carlota Vaz Patto

**Affiliations:** ^1^Instituto de Tecnologia Química e Biológica António Xavier, Universidade Nova de Lisboa, Oeiras, Portugal; ^2^Instituto de Agricultura Sostenible, Consejo Superior de Investigaciones Científicas, Córdoba, Spain

**Keywords:** genome-wide association study (GWAS), grass pea, partial resistance, natural variation, rust

## Abstract

*Uromyces pisi* ([Pers.] D.C.) Wint. is an important foliar biotrophic pathogen infecting grass pea (*Lathyrus sativus* L.), compromising their yield stability. To date, few efforts have been made to assess the natural variation in grass pea resistance and to identify the resistance loci operating against this pathogen, limiting its efficient breeding exploitation. To overcome this knowledge gap, the genetic architecture of grass pea resistance to *U. pisi* was investigated using a worldwide collection of 182 accessions through a genome-wide association approach. The response of the grass pea collection to rust infection under controlled conditions and at the seedling stage did not reveal any hypersensitive response but a continuous variation for disease severity, with the identification of promising sources of partial resistance. A panel of 5,651 high-quality single-nucleotide polymorphism (SNP) markers previously generated was used to test for SNP-trait associations, based on a mixed linear model accounting for population structure. We detected seven SNP markers significantly associated with *U. pisi* disease severity, suggesting that partial resistance is oligogenic. Six of the associated SNP markers were located in chromosomes 4 and 6, while the remaining SNP markers had no known chromosomal position. Through comparative mapping with the pea reference genome, a total of 19 candidate genes were proposed, encoding for leucine-rich repeat, NB-ARC domain, and TGA transcription factor family, among others. Results presented in this study provided information on the availability of partial resistance in grass pea germplasm and advanced our understanding of the molecular mechanisms of quantitative resistance to rust in grass pea. Moreover, the detected associated SNP markers constitute promising genomic targets for the development of molecular tools to assist disease resistance precision breeding.

## Introduction

Grass pea (*Lathyrus sativus* L.) is a cool-season legume crop with considerable economic importance, particularly in the developing nations of India, Bangladesh, and Ethiopia (Dixit et al., 2016; Das et al., 2021). This species is seen as a promising source of calories and proteins, and its resilience to adverse abiotic constraints has great potential for expansion in drought-prone and marginal areas (Vaz Patto and Rubiales, 2014; Rubiales et al., 2020). Grass pea accessions can be classified into two main ecotypes, mostly considering their seed morphological features: one ecotype with accessions with larger and light-colored seeds usually originated from Mediterranean countries, and the other ecotype with accessions with smaller and dark-colored seeds, mostly from Asian countries (Przybylska et al., 2000). The genetic structure analysis was performed using molecular markers of a worldwide germplasm collection of grass pea accessions (Sampaio et al., 2021a). Moreover, further phenotypic characterization revealed that accessions from the Mediterranean region showed higher resistance to *Fusarium oxysporum* Schl. f. sp. *pisi* Snyd. and Hans. (fusarium wilt causal agent in grass pea), as compared with the remaining geographic origins (Sampaio et al., 2021b). These findings highlight the importance of assessing the grass pea germplasm natural diversity for a more educated utilization and conservation of grass pea genetic resources.

Resistance to pests and diseases is an important feature of grass pea (Vaz Patto and Rubiales, 2014) as is for most crops, including legumes (Rubiales et al., 2015). One example of such biotic threats, with a devastating impact on a wide range of legume crops worldwide, is the rust disease (Sillero et al., 2006). Several rust species can infect the Fabaceae family, the majority belonging to the genus *Uromyces* (Link.) Unger (Rubiales et al., 2011). These are biotrophic leaf pathogens, dependent on the infected host cells to remain viable throughout the infection process for successful colonization and completion of the life cycle (Panstruga, 2003; Martins et al., 2020). Both monogenic and polygenic resistances to rust have been reported in common bean, soybean, and faba bean among others, with the identification of loci controlling resistance and the development of closely linked molecular markers for selection breeding (Avila et al., 2003; Miklas et al., 2006; Hyten et al., 2007; Garcia et al., 2008; de Souza et al., 2011; Childs et al., 2018; Ijaz et al., 2021). However, limited information is available on rust resistance in most legumes. Hypersensitive resistance has been reported in lentil for instance (Rubiales et al., 2013), which might suggest monogenic resistance, but genetic analyses are not available so far. In other legumes such as pea or chickpea, hypersensitive resistance has not been identified in spite of thorough searches, although variation for partial resistance (Barilli et al., 2009a; Sillero et al., 2012) and associated QTL have been reported (Madrid et al., 2008; Barilli et al., 2010, 2018). Additionally, a few transcriptomic and proteomic studies on different legume-rust pathosystems indicated that rust induced important molecular changes on a general battery of plant defenses such as pathogenesis-related transcription factors (Madrid et al., 2008; *Medicago truncatula* Gaertn.—*Uromyces striatus*), reactive oxygen species-detoxifying enzymes (Castillejo et al., 2010; *M. truncatula—U. striatus* J. Schröt.), and the phenylpropanoid pathway (Barilli et al., 2015; pea—*Uromyces pisi*).

Previous studies showed that rust in grass pea is mainly attributed to *U. pisi* ([Pers.] D.C.) Wint., the causal agent of pea rust (Vaz Patto and Rubiales, 2009). Partial resistance, characterized by a compatible plant–pathogen interaction (non-hypersensitive response) and reduced disease severity (sensus Parlevliet, 1979), was frequently observed in an Iberian grass pea germplasm collection in response to *U. pisi* infection. Further histological evidence revealed that this partial resistance was attributed to restriction in haustorium formation, reduction in haustorium number per colony, and intercellular growth of infection hyphae (Vaz Patto and Rubiales, 2009).

The genetic architecture of partial resistance is often described to be associated with several loci each with variable effects on the resistance response observed to a pathogen (St. Clair, 2010). This, allied to the observable reduction on pathogen colonization and consequently reduced selective pressure imposed on the pathogen, contributes to increased durability and stability of partial resistance (Pilet-Nayel et al., 2017). This is particularly important for pathogens with a high risk of breaking down resistance genes due to the coexistence of sexual and asexual reproduction systems and with an effective air dispersal, such as rust pathogens (McDonald and Linde, 2002).

A more detailed characterization of the genetic architecture of partial resistance to rust in grass pea is still lacking. This may be in part attributed to the still limited genomic resources available in this species, hampering more efficient exploitation of previously identified resistant grass pea accessions as sources of favorable alleles in breeding for improved resistance. The few efforts taken to revert this unfavorable situation resulted in the development of a high-throughput transcriptome assembly of grass pea accessions (Almeida et al., 2014) and the closely related *Lathyrus cicera* L. accessions (Santos et al., 2018) against *U. pisi* infection. Results from the study by Almeida et al. (2014) revealed that differences among grass pea partially resistant and susceptible accessions were mostly related to the regulation of phytohormones signaling pathways, expression of pathogenesis-related (PR) genes such as the mildew resistance locus O (*mlo*), chitinases involved in fungal cell wall degradation, and genes involved in the production of secondary metabolites with antimicrobial activity. The highlighted pathogenesis-related mechanisms provided a valuable preliminary overview on the resistance mechanism activated in grass pea against rust infection to be further validated with results from other approaches such as genetic mapping.

The availability of cost-effective, high-throughput genome-wide single-nucleotide polymorphism (SNP) genotyping platforms is very relevant, especially in underutilized crops like grass pea (Kamenya et al., 2021). The development of a robust set of molecular markers for a particular species allows fine mapping of linked genomic loci controlling important traits through a genome-wide association study (GWAS). GWAS is a powerful methodology for harnessing the natural variation occurring in plant germplasm collections to dissect the allelic variants controlling complex traits (e.g., partial resistance to biotic stress). This approach has proven successful in better characterizing the response of a worldwide diverse grass pea germplasm collection (the same targeted in this study) against biotic constraints, namely, fusarium blight (Sampaio et al., 2021a). Although the absence of a fully assembled grass pea reference genome imposed some challenges to the interpretation of GWAS results, a comparative approach with the pea reference genome (Kreplak et al., 2019), highly macrosyntenic to grass pea (Santos et al., 2021), has shown to be a reliable strategy to propose candidate genes.

In this study, we searched for novel resistance sources against *U. pisi* in a comprehensive germplasm collection of 182 grass pea accessions representative of worldwide diversity. Furthermore, we studied the genetic architecture of grass pea partial resistance to rust infection, through a GWAS as the first step for a more efficient precision breeding. To achieve this, the phenotypic response of the worldwide grass pea collection of accessions inoculated with *U. pisi* was combined with a previously generated high-throughput SNP markers screening, and significant SNP-trait associations were detected.

## Materials and Methods

### Phenotypic Data

#### Plant Materials and Pathogen Isolates

The disease reaction of a worldwide germplasm collection of 182 grass pea accessions was evaluated in response to *U. pisi*. This germplasm collection was the same as described by Sampaio et al. (2021a) to identify genomic regions controlling resistance for fusarium wilt in grass pea. Two main ecotypes have been defined in grass pea related to their seed morphological traits, mostly composed of accessions with Mediterranean (big and light-colored seeds) or Asian origin (small and dark-colored seeds) (Przybylska et al., 2000). Therefore, as a proxy to this ecotype classification, the germplasm collection was classified based on their seed color (89 accessions with light seed color, and 93 accessions with dark seed color), seed size (52 accessions with large seeds and 130 accessions with small seeds), and geographical origin (91 accessions with European origin, one accession with Canadian origin, one accession with Brazilian origin, 60 accessions with Asian origin, 13 accessions with Ethiopian origin, 10 accessions with North African origin, and six accessions with unknown origin) (Supplementary Table 1). Seed size classification was assigned based on the weight (g) of 100 seeds (below 18 g accessions were considered small, and over 18 g accessions were considered large).

Seeds from the grass pea accessions and the susceptible control pea cv. ‘Messire’ were surface sterilized and germinated as described earlier (Sampaio et al., 2021b). Following this, seedlings were transferred to 0.5 L pots (one plant/pot) containing 1:1 sand and peat mixture and maintained under controlled conditions (12 h light 22°C/12 h dark 20°C photoperiod, 60% relative humidity, and 200 μmol/m^2^ s of illumination). Experiments related to the phenotypic evaluation of the germplasm collection were carried out at the facilities of Consejo Superior de Investigaciones Científicas- Instituto de Agricultura Sostenible (CSIC-IAS), Córdoba, Spain.

The monopustular isolate UpCo-01 of *U. pisi* used in the inoculation assay was kept at −80°C at the Institute for Sustainable Agriculture-CSIC, Córdoba, and was multiplied on plants of the susceptible *Pisum sativum* cv. ‘Messire’ before use.

#### Rust Inoculation and Disease Response Assessment

Grass pea accessions were analyzed using a randomized complete block design. Three independent inoculation assays with *U. pisi* were performed on the 20-day-old whole grass pea plants (3–5 seedlings per accession, per inoculation assay). Inoculations with *U. pisi* were conducted by dusting 2 mg of rust spores/plant diluted with pure talc (1:10, w:w), with the help of a small manual dusting device (Vaz Patto and Rubiales, 2009). Pea cv. ‘Messire’ plants were included as susceptible checks in every inoculation assay. Inoculated plants were incubated for 24 h at 20°C and 100% humidity in complete darkness. Following incubation, spore germination was checked under a microscope, and plants were transferred back to the growth chamber where they were originally maintained. Disease severity (DS) and infection type (IT) were assessed 12 days after inoculation. DS was visually scored as the percentage of leaf area covered by rust pustules. IT was estimated based on the scale of Stakman et al. (1962), where IT 0, no symptoms; IT 1, necrotic flecks with minute pustules barely sporulating; IT 2, necrotic halos surrounding small pustules; IT 3, chlorotic halos surrounding pustules; IT 4, well-formed pustules with no associated chlorosis or tissue necrosis.

#### Phenotypic Data Analysis

Phenotypic data were subjected to residuals inspection to evaluate normality (quantile–quantile plot, QQ), the presence of outliers, and homogeneity of variance (residuals vs. fitted values). Since the residual’s variance followed a normal distribution, no data transformation was applied.

A linear mixed model was applied for the DS trait, DS = *accessions* + *inoculation assay* + *accessions.inoculation assay*, where *accessions* is the genotypic term, *inoculation assay* (1–3) corresponds to the three independent inoculation assays, and *accession.inoculation assay* corresponds to the interaction between accessions and the independent inoculation assays. In a first step, best linear unbiased predictors (BLUPs) were obtained while fitting the model with all terms as random. A restricted maximum likelihood (REML) was applied to estimate the variance components of the linear mixed model and broad-sense heritability [VHERITABILITY procedure in Genstat software, according to Cullis et al. (2006)]. Following this, the best linear unbiased estimates (BLUEs) for each accessions were estimated while setting the term *accession* as fixed and the terms *accessions*.*inoculation assay* and *inoculation assay* as random. A Wald test for the significance of the fixed effects was performed using the generated BLUEs dataset.

A linear mixed model was used to estimate how much of the variation of accessions’ response to *U. pisi* infection could be explained by geographical origin or seed morphology (seed color and size). The following linear mixed model was applied to investigate differences among seed origin classes: DS = *seed origin* (fixed term) + *inoculation assay* + *accessions* (random terms). A similar model was used to estimate how much of the accessions’ variance was explained by *seed size* or *seed color*, replacing *seed origin* with each of these terms. A Wald test was performed to test the significance of the fixed effects. Fisher’s multiple-comparison tests were applied to the means of DS scores, at *P*-value ≤ 0.05. All analyses were performed using the Genstat software, 20th edition (VSN International, 2021).

### Genotypic Data

#### Association Mapping Analysis

A GWAS was performed with GenStat software in the mixed-model framework, fitting SNP markers as fixed terms and accessions as random terms, using REML (Malosetti et al., 2007). Adjusted means (BLUEs) of the DS scores from the 182 grass pea accessions were tested for association with a previously available genotypic dataset.

Both the genotypic datasets constituted 5,651 SNP markers after quality control, and the pea genome marker positions previously described by Sampaio et al. (2021a) were retrieved to perform the present association analysis. This genotypic dataset was obtained from two high-throughput genotyping-by-sequence providers [Dart-Seq™ genotyping (Kilian et al., 2012) and BGI, Beijing Genomic Institute, Beijing, China] using genomic DNA extracted from young leaves. Physical positions of SNP markers were assigned based on the pea reference genome v1a (Kreplak et al., 2019) as the most phylogenetic closely and highly syntenic-related species (Santos et al., 2021) with a better assembled sequenced genome.

Three linear mixed models, as described in the study of Sampaio et al. (2021a), were tested to control for false-positive SNP-trait associations: a naïve model, not accounting for population structure or family relatedness (Phenotype = SNP + *Error*); a model accounting for population structure (Q), using 15 principal components from the principal component analysis (PCA) (*Phenotype* = Q + *SNP* + *Error*); and a model accounting for familial relatedness (*K*), using kinship matrix *K* (*Phenotype* = *SNP* + *Accession* + *Error*), with *Accession* random effects structured following a kinship matrix *K* (Malosetti et al., 2007).

The principal components to account for the population structure among accessions and the kinship matrix to account for familial relatedness among accessions were previously calculated by Sampaio et al. (2021a) and retrieved to use in the present analysis. These calculations were made using a total of 1,058 SNP markers, evenly distributed across the pea genome, corresponding approximately to 1 SNP per megabase pair (Mbp).

The most appropriate model was selected following the inspection of the inflation factor value and quantile–quantile (QQ) plots of the *P-*values with the least deviations from the null hypothesis. The observed –log_10_(*P-*value) of each SNP marker was plotted against their assigned pea chromosomal position, based on comparative mapping with the pea reference genome v1a (Kreplak et al., 2019), to generate a Manhattan plot. Significant SNP-trait associations were detected at a threshold of –log_10_(*P-*value) = 3.5. This threshold was established taking into consideration two aspects: the size of the association panel used and the background noise of the Manhattan plots. Similar criteria were already described in other works with comparable or slightly smaller panel sizes and a similar number of markers, focusing on partial resistance traits (Tessmann and Van Sanford, 2018; Basile et al., 2019; Leitao et al., 2020) to avoid losing potentially interesting regions while applying a conservative type of adjustment such as Bonferroni correction [–log_10_(*P-*value) = 5.053]. Moreover, adjusted *P*-values according to the Benjamin and Yekutieli false discovery rate procedure (Benjamini and Yekutieli, 2001) were calculated considering an α = 0.2 and *k* (number of LD blocks per chromosome) = 3,007, to control for type I errors caused by multiple testing. The effect of the minor frequency SNP allele was estimated in relation to the most frequent reference allele.

#### Allelic Variant Frequency on the Single-Nucleotide Polymorphisms Associated With the Trait of Interest Within Seed Classes

Frequencies of the favorable allele contributing to resistance, of each SNP marker detected as associated with the DS trait in response to *U. pisi* inoculation, were calculated by counting the number of accessions with a given seed color (dark or light), seed size (small or large), and geographical origin (Canadian, Brazilian, Asian, Ethiopian, European, North African or with unknown origin) that had the favorable allelic variant and divided by the total number of accessions with the same seed trait or geographical origin.

#### Linkage Disequilibrium and Candidate Gene Identification

Linkage disequilibrium (LD) per chromosome was estimated as the squared coefficient of correlation between marker pairs (*r*^2^), after correction for population structure using the principal component scores from Eigenanalysis as implemented in Genstat software and described by Sampaio et al. (2021a). For this calculation, all grass pea markers with an assigned position (3,180 SNP markers) on the pea reference genome were considered. LD decay was visualized for each chromosome while plotting *r*^2^ against the physical mapping distance in Mb. The LD decay threshold (*r*^2^ = 0.2) was used to estimate the average genetic distance for which markers were considered to be no longer correlated. Accordingly, the distances to which LD decayed to the *r*^2^ threshold for the chromosomes where SNP-trait associations were detected are the following: 0.074 Mb for chromosome 4 and 0.14 Mb for chromosome 6.

A genomic window for each SNP marker location significantly associated with the trait measured was established by subtracting and adding the average genetic distance considering the respective chromosomal LD decay. The physical boundaries of each chromosomal LD block (for which LD *r*^2^ > 0.2) were used as query positions on the pea reference genome v1a (Kreplak et al., 2019) to retrieve the list of candidate genes mapped within those boundaries. Candidate genes for the response to *U. pisi* were considered if they contained a significantly associated SNP or were in LD with a significantly associated SNP marker. Annotation of the candidate genes was given by the JBrowse tool available at https://urgi.versailles.inra.fr/Species/Pisum. Candidate gene functional characterization was obtained using the Mercator v2.0 (Schwacke et al., 2019).^1^

### Candidate Gene Relative Expression Analysis by Reverse-Transcribed Quantitative PCR

#### Plant Material, RNA Extraction, and cDNA Synthesis

Three partially resistant grass pea accessions (i.e., PI165528 DS = 20%, PI283566 DS = 12%, and PI577183_A DS = 23%) and two susceptible accessions (i.e., PI283574 DS = 30% and PI221467_B DS = 38%) to *U. pisi* infection were selected for gene expression of candidate genes Psat6g006240 and Psat4g145320 (harboring the SNP markers detected as associated with the disease response), plus the candidate gene Psat6g010840 (in LD with SNP2174 and SNP2175). Three plants per accession (biological replicates) per time-point were inoculated as described earlier (see the “Materials and Methods” section). Leaves were collected from non-inoculated plants at 0 h after inoculation (HAI) and from inoculated grass pea plants at different time points (e.g., 12 HAI, 24 HAI, and 48 HAI). Leaves were immediately frozen in liquid nitrogen and stored at −80°C until RNA extraction.

Total RNA was extracted from 100 mg of frozen leaves grounded to a fine powder in liquid nitrogen using a mortar and a pestle. RNA was isolated using the GeneJet Plant RNA Purification Kit (ThermoFisher Scientific, Vilnius, Lithuania) and treated with DNase I Kit (Ambion, Austin, TX, United States). RNA quantification was performed using Qubit 2.0 Fluorometer (Life Technologies, NY, United States) with Qubit RNA BR Assay Kit (ThermoFisher Scientific, Waltham, MA, United States). RNA purity was assessed by wavelength ratios measurement (260/280 and 260/260 nm) using Nanodrop ND-2000C spectrophotometer (ThermoFisher Scientific, Waltham, MA, United States). cDNA was synthesized from 500 ng of total RNA from each sample using the iScript cDNA Synthesis Kit (Biorad, Hercules, CA, United States).

#### Primer Design

Specific primers were designed for the target candidate genes using the gene sequence obtained with the JBrowse tool^2^ as a template. The Primer3Plus tool^3^ (Boston, United States) was used for primer design, with the default setting for RT-qPCR optimal conditions, and primer specificity was assessed using the Primer-BLAST NCBI tool. Specific primers were designed in the 3′ intra-exonic regions and synthesized by STABVida (Caparica, Portugal). Primer sequences can be found in Supplementary Table 2.

#### Quantitative Reverse-Transcribed Quantitative PCR Assay

Relative gene expression of target candidate genes among partially resistant and susceptible grass pea accessions was analyzed by RT-qPCR on a Light Cycler^®^ 480 System, using the LighCycler 480 SYBR Green I Master protocol. As reference genes β-tubulin, photosystem I P700 apoprotein, y-tubulin, helicase, and histone H2A.2, previously described as reference genes for *Lathyrus* spp. (Almeida et al., 2014; Santos et al., 2018), were tested. Using the geNorm and NormFinder packages from GenEx v.5 software (MultiD, Goteborg, Sweden), histone H2A.2 and *y-*tubulin were selected as reference genes for the following gene expression assays.

Reverse-transcribed quantitative PCR (RT-qPCR) was performed for each of the three biological replicates per accession and time-point assessed (non-inoculated, 12 HAI, 24 HAI, and 48 HAI). Thermo cycling reactions were carried out following the described conditions: denaturation step at 90°C for 5 min; 45 cycles of amplification for 10 s at 95°C; 10 s at 60°C, and 10 s at 72°C. A melting curve was performed to detect non-specific PCR products or contaminants. A non-template control without cDNA was included for each primer mix to detect possible contaminations.

Relative expression values of each candidate gene were normalized to both reference genes using as calibrator the relative expression values of the non-inoculated (0 HAI) samples of the most susceptible accession to PI221467_B, following the Pfaffl (2001) method (2001). Fold change data were transformed into a logarithmic scale (base 2). A two-way ANOVA was conducted to inspect for differences between accessions and time-points per candidate gene. The *post hoc* Tukey’s multiple comparison tests were used for means comparison at *P*-value ≤ 0.05.

## Results

### Continuous Variation of Resistance Response in Grass Pea Response to *Uromyces pisi* Inoculation Might Be Related to Geographical Origin but Not to Ecotype Classification

**FIGURE 1:**
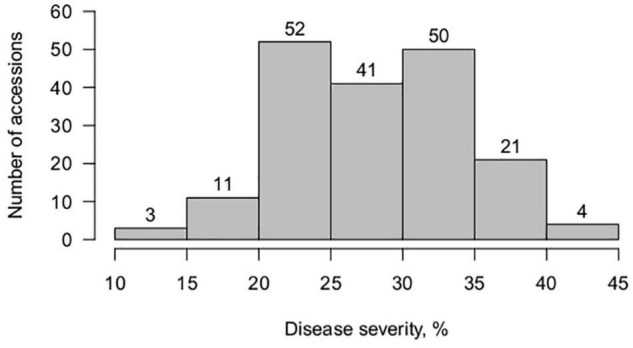
Frequency distribution of *Uromyces pisi*-induced disease severity (DS) scores (%) in a worldwide collection of 182 grass pea accessions.

All grass pea accessions showed a compatible interaction with *U. pisi*, with no associated macroscopically visible necrosis on the leaf surface (IT 3-4). In spite of this high IT, a continuous variation was observed in terms of disease severity, from 10 to 45%, with an average of 28.2 ± 6.3%, with significant differences detected among accessions (*P*-value ≤ 0.05, Wald test). The majority of the studied accessions were moderately susceptible (DS > 25%) to the *U. pisi* isolate used in this study (Figure 1). None of the grass pea accessions exhibited complete resistance (total absence of symptoms). Reduced DS scores (DS < 25%), in spite of high infection type (e.g., IT 4 and IT 3), were detected in 35% of the germplasm collection, hinting for partial resistance. Broad-sense heritability of grass pea DS for *U. pisi* was 0.89 (Supplementary Table 3).

Accessions with Ethiopian origin were shown to be the most susceptible (higher DS values) to rust infection (*P* ≤ 0.05). Moreover, when accessions were classified based on seed color (dark or light), dark seed color accessions were the most susceptible. As for accessions classified by seed size, no differences were observed among larger or smaller seeds (Supplementary Table 4).

### Grass Pea Partial Resistance to *Uromyces pisi* Is Controlled by Multiple Loci

Adjusted means calculated from the DS scores of 182 accessions were tested for SNP-trait associations with the previously generated high-quality 5,651 SNP markers, 2,471 of which had no known chromosomal position based on comparative mapping with pea reference genome v1a (Kreplak et al., 2019).

As previously described for the grass pea association panel understudy (Sampaio et al., 2021a), a clear genetic structure was detected, which, if not accounted for, could lead to the detection of false-positive associations. For this reason, SNP-trait associations were tested comparing a linear mixed model not accounting for any structure (naïve model) with models considering either population structure (Eigenanalysis) or kinship relationship among accessions (*K* matrix). Following an inspection of inflation factor values (near 1 is indicative of a better-fitting model) (Supplementary Table 5) and Q–Q plots (Supplementary Figure 1) of the *P-*values with the least deviations from the null hypothesis of the models tested, the model accounting for population structure (Eigenanalysis) was selected as the most appropriate. Results described hereafter were obtained with this model.

**FIGURE 2:**
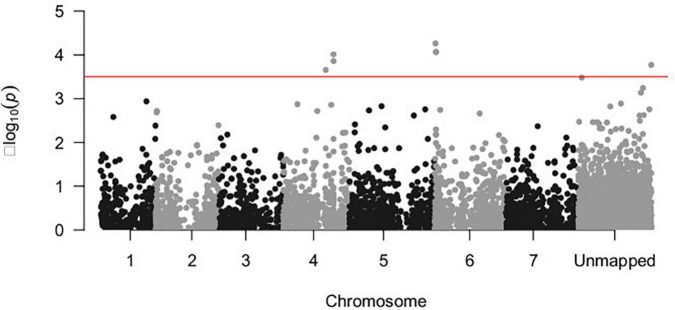
Manhattan plot depicting the –log_10_(*P*-value) vs. chromosomal position of 5,651 SNP markers associated with the disease response of a grass pea collection of 182 accessions infected with *U. pisi*. The red line shows the threshold –log_10_(*P*-value) = 3.5 for the detection of significantly associated genomic regions.

A total of seven SNP markers were significantly associated with the response to *U. pisi* inoculation (measured by DS) using a threshold of −log_10_(*P-*value) = 3.5 (Figure 2). Six of the associated SNP markers were located in chromosomes 4 and 6, while the remaining SNP markers had no known chromosomal position. SNP2145, located on chromosome 6, had the strongest association with the DS trait [−log_10_(*P*-value) = 4.262]. Each of the associated SNP markers explained only a portion of the observed phenotypic variance (6.5–8.1%). The SNP markers that explained the biggest proportion of phenotypic variation were SNP2174 (7.8%), SNP2175 (7.8%), SNP2145 (8%), and SNP1323 (8.1%) (Table 1).

**TABLE 1:** List of single-nucleotide polymorphism (SNP) markers significantly associated [−log_10_(*P-*value) = 3.5] with grass pea response to *Uromyces pisi* infection.

SNP ID	Pea Chr	−log_10_(*P-*value)	*P* value	Adjusted by *p^a^*	Reference allele	Variant allele	Frequency*^b^*	Effect*^c^*	*V*_*QTL*_/*V*_*G*_*^d^*%
SNP1323	4	3.655	2.2 × 10^–4^	1.4 × 10^–4^	C	G	0.4444	−2.0284	8.1
SNP1385	4	3.860	1.3 × 10^–4^	1.7 × 10^–4^	A	G	0.0675	−3.5784	6.5
SNP1402	4	4.012	9.7 × 10^–5^	1.7 × 10^–4^	C	T	0.0915	−3.2601	7.1
SNP2145	6	4.262	5.4 × 10^–5^	2 × 10^–4^	A	C	0.1234	−3.0442	8
SNP2174	6	4.064	8.6 × 10^–5^	1.8 × 10^–4^	A	G	0.2532	−2.2660	7.8
SNP2175	6	4.064	8.6 × 10^–5^	1.8 × 10^–4^	A	C	0.2532	−2.2660	7.8
SNP5649	Unmapped	3.771	–	–	G	A	0.057	3.9820	6.8

*For each SNP locus, the chromosomal position through comparative mapping with pea genome v1a, the effect of the variant allele, and the proportion of genotypic variance explained are shown.*

*^a^Calculated according to Benjamin and Yekutieli procedure.^b^Frequency of the variant allele.*

*^c^Effect of the variant allele.*

*^d^Proportion of the genotypic variance explained by each of the significantly associated SNPs [V_QTL_ = 2Freq (1 − Freq) effect2; V_G_ = Estimated variance of the genotypic component].*

A negative effect on grass pea DS of the variant allele in relation to the most frequent allele was detected for the majority of the SNP-trait associations, the exception to this being SNP5649. Given that DS is a measure of susceptibility, a negative effect of the variant allele suggests that the presence of the mentioned allele promotes increased resistance to rust infection.

### Accessions With European and North African Origin Are Promising Sources of Allelic Variants Contributing to Partial Resistance

**FIGURE 3:**
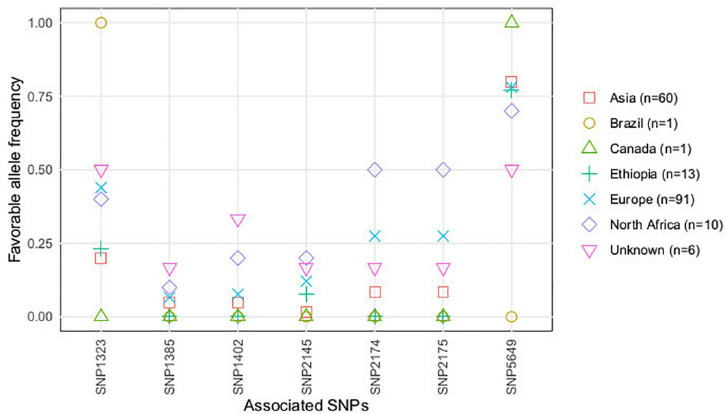
Favorable alleles’ frequency (conferring resistance) of the SNPs associated with *U. pisi* DS, based on the grass pea accessions classified by seed size (large, small) and seed color (light, dark).

According to the accessions’ geographical origin, favorable alleles of the significantly associated SNPs, contributing to partial resistance against *U. pisi* infection, showed higher frequencies in accessions with North African, European, and with unkown origin (Figure 3). A different situation was observed for SNP5649, presenting a higher frequency of favorable alleles in accessions with Asian, Ethiopian, and American (Canadian and Brazilian) geographical origins.

**FIGURE 4:**
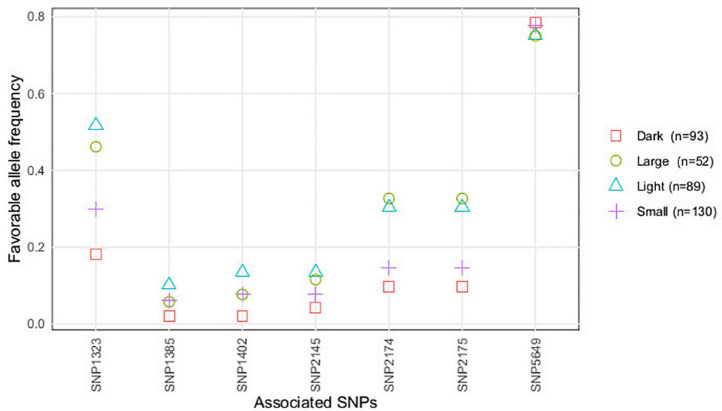
Favorable allele’s frequency (conferring resistance) of the SNPs associated with *U. pisi* DS, based on the grass pea accessions classified by geographical origin.

When accessions were classified based on their seed color (light or dark) and seed size (large or small), we observed that allelic frequencies of accessions with light-colored and larger seeds matched being the same observed for accessions characterized with dark-colored and smaller seeds (Figure 4). For SNP1323, SNP2174, and SNP2175, favorable allele frequencies were highest for accessions with light and larger seeds, whereas for the remaining SNPs almost no differences were detected between the seed color and seed size classes of accessions.

For the majority of the associated SNPs’ favorable alleles (conferring resistance), a decrease in allele frequency was observed in accessions with increasing DS scores (Supplementary Figure 2). SNP5649 was the exception to this, presenting the highest frequency of the favorable allele conferring partial resistance, still present in accessions with a DS score over 40%.

### Partial Resistance Candidate Genes Are Involved in a Diverse Array of Cellular Functions

Considering an LD decay threshold of *r*^2^ = 0.2, the associated SNP markers SNP1385 and SNP1402 were in LD. The same was observed for the SNP2174 and SNP2175, sharing the same chromosomal region. The chromosomal locations of the significantly associated SNP markers detected through GWAS were inspected to propose candidate genes using comparative mapping with pea reference genome v1a (Kreplak et al., 2019). The genomic window selected to search for candidate genes was established considering the LD decay of the chromosome where the SNP-trait association was detected. Candidate genes harboring, or in LD, with associated SNP markers with known chromosomal position were identified following SNP marker sequence alignment against the pea genome. Accordingly, a total of 19 candidate genes were highlighted. A list with all the candidate genes can be found in Supplementary Table 6. Except for SNP1385 and SNP1402, all SNP markers significantly associated with the disease response to *U. pisi* were mapped within genes. Moreover, considering the degree of chromosomal LD block around each SNP marker identified, we achieved a mapping resolution to the gene level in 50% of the cases (2 LD blocks where a single candidate gene was identified).

Considering the multiple candidate genes linked to the detected SNP-trait associations, we will restrict the candidate genes description to those that were detected harboring the most strongly associated SNP marker and with biological relevance in the context of disease resistance. The strongest significant association [SNP2145 on chromosome 6, −log_10_(*P-*value) = 4.262] was mapped within the protein-coding gene Psat6g006240, coding for the GTP1/OBG GTP-binding protein family signature. In LD with SNP2145, the candidate gene Psat6g006320 was identified. This gene codes for an NB-ARC domain, the key regulator of the activity of resistance (R) proteins. SNP2174 and SNP2175 (chromosome 6), also among the strongest significant associations [−log_10_(*P*-value) = 4.064], were located within the coding sequence of Psat6g010800, a gene coding for the multidrug and toxin extrusion (MATE), and required for the transport of secondary metabolites and phytohormones, among others. Considering the LD decay in the chromosome where these SNP markers were mapped, a leucine-rich repeat (LRR) domain, known structural features of the majority of plant R proteins, was identified as a candidate gene.

Besides the identified candidate genes linked to the regions where the strongest SNP-trait associations were detected, an additional candidate gene putatively involved in disease resistance pathways was identified. On chromosome 4, SNP1323 was located within the gene Psat4g145320. This gene codes for a seed dormancy control protein, functionally annotated as the basic leucine zipper (bZIP) TGA transcription factor (TF), known to regulate the expression of pathogenesis-related (*PR*) genes.

### Reverse-Transcribed Quantitative PCR Results Highlight Psat6g006240 (GTP1/OBG GTP-Binding Protein Family Signature) and Psat6g010840 (Leucine-Rich Repeat) as Putative Candidate Genes Differentially Expressed Among Grass Pea Accessions With Contrasting Rust Infection Responses

Relative gene expression of Psat4g145320 (bZIP TGA transcription factor) and Psat6g006240 (GTP1/OBG GTP-binding protein family signature) selected as candidate genes harboring significantly associated SNP markers, and Psat6g010840 (leucine-rich repeat) in LD with SNP2174 and SNP2175, were analyzed by RT-qPCR in phenotypically contrasting grass pea accessions (partial resistant vs. susceptible). Expression analysis of the candidate gene Psat6g010800 was not conducted given the struggle found in designing specific primers, and thus, no gene expression data are presented for the mentioned gene. Patterns of gene expression at different time-points (non-inoculated, 12 HAI, 24 HAI, and 48 HAI) among grass pea accessions with contrasting disease responses are presented in Supplementary Figure 3.

Regarding candidate gene Psat6g006240, we detected a significant gene downregulation in the susceptible accession (PI283574), as compared with the partially resistant accessions (e.g., PI283566, PI577138_A, and PI165528). In the susceptible accession, candidate gene downregulation was observed by 12 HAI (fold change > 1), culminating at 48 HAI with a fold change of 2, whereas in the partially resistant accessions, gene expression was unchanged throughout the infection process (fold change < 1) (Supplementary Figure 3A). In the candidate gene Psat6g010840, differences in relative gene expression were observed while comparing the partially resistant (PI577138_A and PI165528) and the most susceptible accession (PI221467_B). In the partially resistant accessions, we observed a constitutive relative gene expression throughout the time-points investigated, whereas in the susceptible accession, a continuous increase in relative gene expression was detected, culminating at 48 HAI with a 1-fold change (Supplementary Figure 3B). As for the remaining candidate gene (Psat4g145320), no significant differences were detected among the phenotypically contrasting grass pea accessions.

## Discussion

Although considered a model species for a more sustainable agriculture due to its resilience to different stresses, little is known about the genetic basis of disease resistance, particularly to rust infection, in grass pea. This has restricted an efficient use of existing sources of resistance in grass pea precision breeding. To address this knowledge gap, we targeted through a GWAS a comprehensive germplasm collection of 182 grass pea accessions representative of worldwide diversity, revealing different sources of partial resistance to *U. pisi* infection. Several identified associated genomic regions suggested an oligogenic control of rust resistance in grass pea and allowed the proposal of potential candidate genes.

A continuous variation in the disease response to *U. pisi* was observed on the grass pea collection of accessions characterized here. This variation encompassed a wide range of responses from partially resistant to highly susceptible accessions. The most frequently observed phenotype was a compatible interaction (IT 3, IT 4), not associated with hypersensitive reaction, as it has been previously described on a more regional collection of Iberian grass pea accessions (Vaz Patto and Rubiales, 2009), and fitting the partial resistance definition (Parlevliet, 1979). In other legume-rust pathosystems, incomplete non-hypersensitive type of resistance was also the most frequently observed. In a very similar manner, a continuous distribution of disease response to *U. pisi* was observed in a pea germplasm collection, and the partially resistance sources identified were non-hypersensitive (Barilli et al., 2009b). This was also the case in chickpea (*Cicer arietinum* L.)—*Uromyces ciceris-arietini* Grognot (Sillero et al., 2012).

Moreover, the described natural variation of grass pea response to *U. pisi* resulted in the identification of seven significantly associated SNP markers, distributed by at least two different chromosomes. This demonstrates that the analyzed variation is controlled by multiple loci. This oligogenic nature of resistance to rusts has been often described in cool season legumes (Barilli et al., 2010, 2018; Rai et al., 2011; Childs et al., 2018). The partial resistant phenotypes of the grass pea described here, allied to the polygenic basis of resistance mapped in this study, are particularly interesting in the context of promoting more durable and stable crop protection in plant breeding for pathogen control, particularly for rust pathogens that, due to their effective air dispersal and co-existence of sexual/asexual reproduction strategies, are among the pathogens with increased risk of breaking down the effectiveness of resistance genes (McDonald and Linde, 2002).

Each of the significantly associated SNP marker identified here explained a fraction of the total genotypic variation, ranging from 6.5 to 8.1%. The multiple SNP-trait associations identified, allied to the reduced effect of each SNP marker on the trait measured, are consistent with the partial and quantitative nature of the grass pea response to *U. pisi.* Considering the high broad-sense heritability (0.89), this suggests that additional molecular components of disease resistance to *U. pisi*, explaining the remaining genetic variance, remained to be identified. It could be that a myriad of common variants of smaller effects (as usually occurs in polygenic traits, St. Clair, 2010) are contributing to the measured variance, but they were not uncovered by this association mapping approach. Another factor that could explain this missing heritability could be related to the occurrence of additional causal rare variants that could further contribute to the genotypic variance in the association panel (Eichler et al., 2010). However, given the adopted minor allele frequency (<5%) threshold for SNP data quality control, due to the limited statistical power to detect their contribution to the natural phenotypic variation (Sham and Purcell, 2014), rare allelic variants were excluded from the association mapping analysis.

Several of the significantly associated SNP markers detected here were located within or in LD with *a priori* candidate genes putatively involved in disease resistance pathways. The identification of such biologically relevant candidate genes further strengthens the usefulness of the GWAS approach to better understand the genetic basis of partial resistance to *U. pisi* in grass pea. For instance, in LD with SNP2145 [the strongest association with the DS measured, −log_10_(*P-*value) = 4.262] we identified an NB-ARC domain coding gene (Psat6g006320). This domain is a signaling motif shared by the nucleotide-binding leucine-rich repeat (NB-LRR) protein family, regarded as key R proteins conferring resistance to a wide variety of plant pathogens (Tameling and Joosten, 2007). Structure-function analysis on the NB-ARC domain highlighted their regulatory role in controlling NB-LRRs’ activity (Van Ooijen et al., 2008). Another important common domain among several R proteins, LRR coding gene was also identified (Psat6g010840) in LD with SNP2174 and SNP2175. This domain is believed to provide recognition specificity for pathogen-derived elicitors (Tameling and Joosten, 2007). Although often associated with the expression of complete resistance, *R* genes have also been detected as co-localized with QTL controlling partial resistance in other pathosystems (Kump et al., 2011; Fukuoka et al., 2015; Raboin et al., 2016). Previous studies have hypothesized that functional polymorphisms on R proteins can alter disease resistance in a quantitative manner and contribute to continuous phenotypic variation among accessions in an association panel (Fukuoka et al., 2015). Contrasting with its role in disease resistance, our RT-qPCR results of the Psat6g010840 showed an induced gene expression upon infection with *U. pisi* in the susceptible accession (PI221467_B), culminating at 48 HAI (1-fold change). As for the partially resistant accessions (PI577138_A and PI65528), the candidate gene showed a significantly lower constitutive expression. This suggests that Psat6g010840-induced expression might be contributing to disease susceptibility upon rust infection in grass pea, which seems to contradict their involvement in plant immunity as described above. Another important protein family related to the significantly associated SNP markers (SNP1323) is the basic leucine zipper (bZIP) TGA transcription factor (TF), coded by the candidate gene Psat4g145320. These TFs are required for SA-dependent plant defense responses effective against biotrophic pathogens and known to regulate the expression of *PATHOGENESIS-RELATED* (*PR*) genes (Wang et al., 2016). Despite their biological relevance, we detected no obvious gene expression patterns among phenotypically contrasting grass pea accessions for this candidate gene.

Co-localized with SNP2145, we identified the candidate gene Psat6g006240 (coding for GTP1/OBG GTP-binding protein family signature), differentially expressed among grass pea accessions with contrasting phenotypes to *U. pisi* infection. RT-qPCR analysis on the mentioned candidate gene revealed a significant downregulation in the more susceptible accessions (PI283574) following *U. pisi* infection (fold change > 1 from 12 HAI onward), whereas in the partially resistant accessions (PI283566, PI577138_A, and PI165528), gene expression remained unchanged throughout the time-points assessed (fold change < 1). The basic functions of this subfamily of proteins in plants are not well described, particularly their putative function in contributing to disease resistance. Although this finding might hint at a possible role of the mentioned candidate gene in resistance to rust infection, a more detailed characterization, by functional validation and analysis of global transcriptomic profile, is still required.

Until this study, there was a lack of studies focusing on the identification of resistance genes effective against *U. pisi* infection in legumes overall. This situation compromises efforts to assess if the SNP-trait associations identified here could harbor *a priori* resistance genes with particular relevance for *U. pisi* infection, which could be mapped through comparative mapping with, for instance, the pea reference genome used in this study. Indeed in the case of pea, previous efforts to map genomic regions controlling *U. pisi* resistance were developed in the wild-related *Pisum fulvum* Sibth. and Sm. and resulted in the identification of three QTLs: *UpDSII* (assigned to pea chromosome 4), *UpDSIV*, and *UpDSIV.2* (assigned to pea chromosome 6) (Barilli et al., 2018). When analyzing the pea genomic position of the molecular markers mapped in the proximity of the mentioned QTLs, we identified several SNP markers included in our genotypic panel that were flanking the *UPDSIV* QTL. However, they were not detected as significantly associated with the DS presently measured.

Previous studies have highlighted the usefulness of comparative mapping with the pea reference genome to propose resistance loci in grass pea. This has been further supported by the recent work of Santos et al. (2021), describing a high linkage map synteny between grass pea and pea. Nonetheless, in this study, 1 of the 7 significantly associated loci was considered unmapped, due to lack of alignment with the pea reference genome (considering the threshold *E*-value < 1 × 10^–5^), hindering the proposal of a candidate gene. These constraints highlight the need for a fully assembled and annotated grass pea reference genome (still ongoing, Emmrich et al., 2020) to attain the full potential of GWAS in the identification of candidate genes of interest and to infer about the molecular components encoded by them. The release of a fully annotated grass pea reference genome would lead to a higher percentage of SNP markers with a known chromosomal position and to identify further candidate genes. Nevertheless, it was still possible to get extra evidence on the relevance of the unmapped associated SNP marker on the genetic control of the grass pea—*U. pisi* pathosystem. Indeed, the marker sequence of the unmapped associated SNP marker identified herein (SNP5649) had high sequence homology with one contig found differentially expressed in the RNA-Seq transcriptomic comparison between resistant and susceptible grass pea accessions against *U. pisi* inoculation (Almeida et al., 2014). This contig (a423210_6) was detected to be over-expressed in the resistant accession as compared with the susceptible (fold change > 1), but unfortunately with no functional annotation yet assigned.

Previous genetic diversity studies on the association panel analyzed here have structured the collection in two genetic clusters: one mostly consisting of accessions with lighter and larger seeds, mainly from Europe and North Africa, while darker and smaller seed accessions, predominantly from Asia, composed a second cluster (Sampaio et al., 2021b). In this study, we observed that dark-colored seed accessions, with Ethiopian origin, were the most susceptible against *U. pisi*, as compared with the remaining accessions. Accessions originated from North Africa with light and large seeds had a high frequency of the favorable alleles for the majority of the associated SNPs. This observation reveals that the mentioned grass pea accessions could constitute a promising source of resistant alleles, especially for breeding efforts focused on varieties with similar seed morphological types. Accessions with Canadian and Brazilian origin also presented high frequencies of the favorable allele for SNP1323 and SNP5649; however, these results might be overestimated given the fact that these geographic groups were composed of only one accession each.

The results presented here highlight the usefulness of exploiting the natural variation in grass pea germplasm to reveal genomic regions controlling resistance to *U. pisi*. To the best of our knowledge, this is the first study on association mapping of partial resistance in grass pea *U. pisi* pathosystem. We observed a continuous range of disease responses in response to rust infection. Several newly resistant loci controlling partial resistance to *U. pisi* and putative *a priori* known and novel resistance genes were identified, suggesting an oligogenic basis; however, detailed functional characterization is needed to better describe the underlying molecular mechanisms. Nevertheless, the identified favorable SNP alleles constitute already important genomic tools to assist precision resistance breeding initiatives in grass pea and phylogenetically related legume crops such as pea.

## Data Availability Statement

The datasets presented in this study can be found in online repositories. The names of the repository/repositories and accession number(s) can be found in the article/Supplementary Material.

## Author Contributions

DM carried out the inoculation assays, analyzed the phenotypic data, performed the genome-wide association study and RT-qPCR experiments, and drafted the manuscript. DR participated in the inoculations and discussion of the results and revised the manuscript critically. MCVP coordinated the study and took part in the discussion, drafting, and revision of the manuscript. All authors contributed to the article and approved the submitted version.

## Conflict of Interest

The authors declare that the research was conducted in the absence of any commercial or financial relationships that could be construed as a potential conflict of interest.

## Publisher’s Note

All claims expressed in this article are solely those of the authors and do not necessarily represent those of their affiliated organizations, or those of the publisher, the editors and the reviewers. Any product that may be evaluated in this article, or claim that may be made by its manufacturer, is not guaranteed or endorsed by the publisher.

## Footnotes

1
https://www.plabipd.de/portal/mercator4

2
https://urgi.versailles.inra.fr/Species/Pisum

3
https://primer3plus.com
