# Supplementary material for: Association Mapping of Lathyrus sativus Disease Response to Uromyces pisi Reveals Novel Loci Underlying Partial Resistance
Source: Front Plant Sci. 2022 Mar 24;13:842545. doi: 10.3389/fpls.2022.842545 (PMC8988034; doi:10.3389/fpls.2022.842545)
Supplement: Supplementary file 1 [file Data_Sheet_1.docx]

Supplementary Material


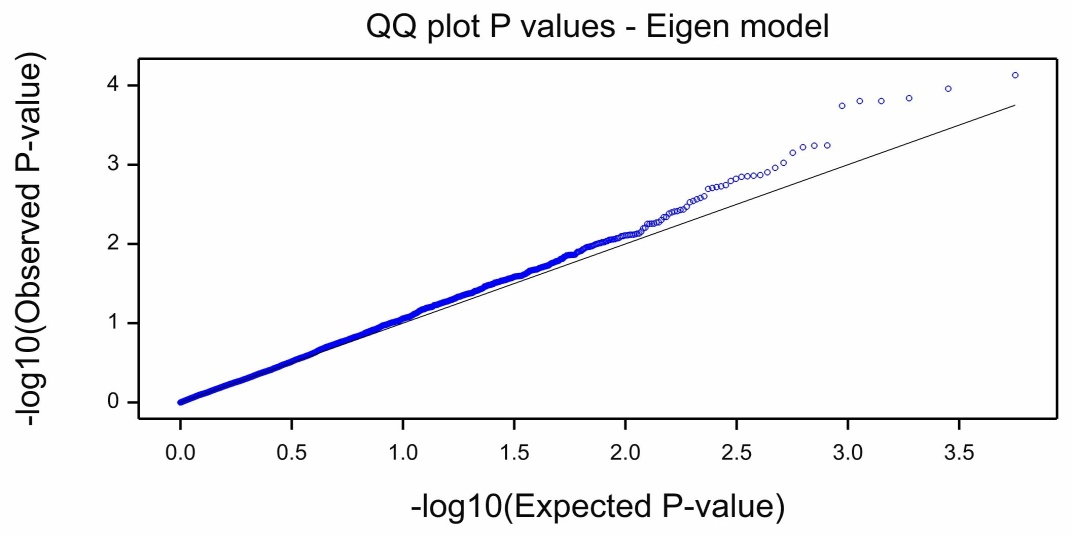

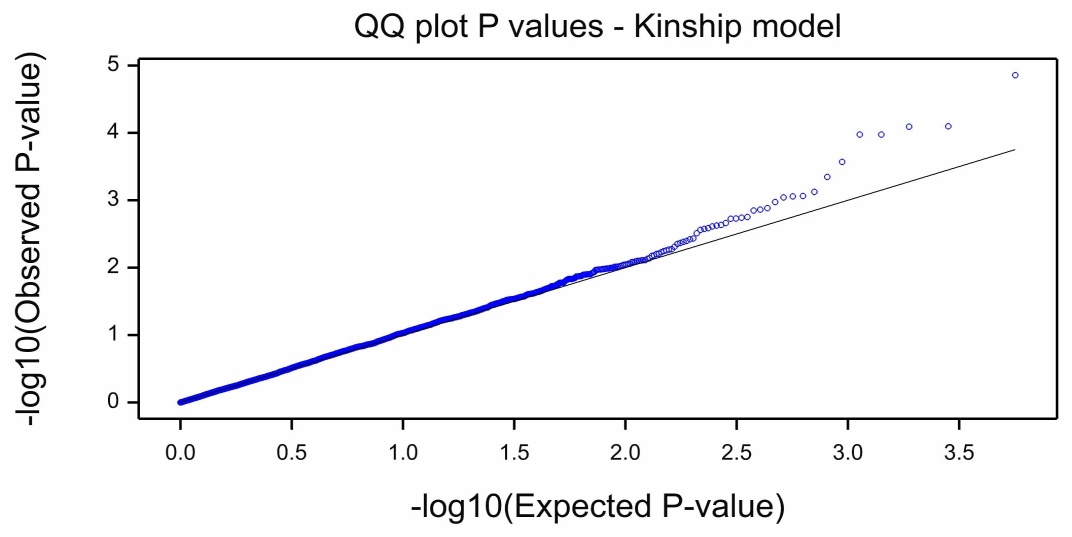


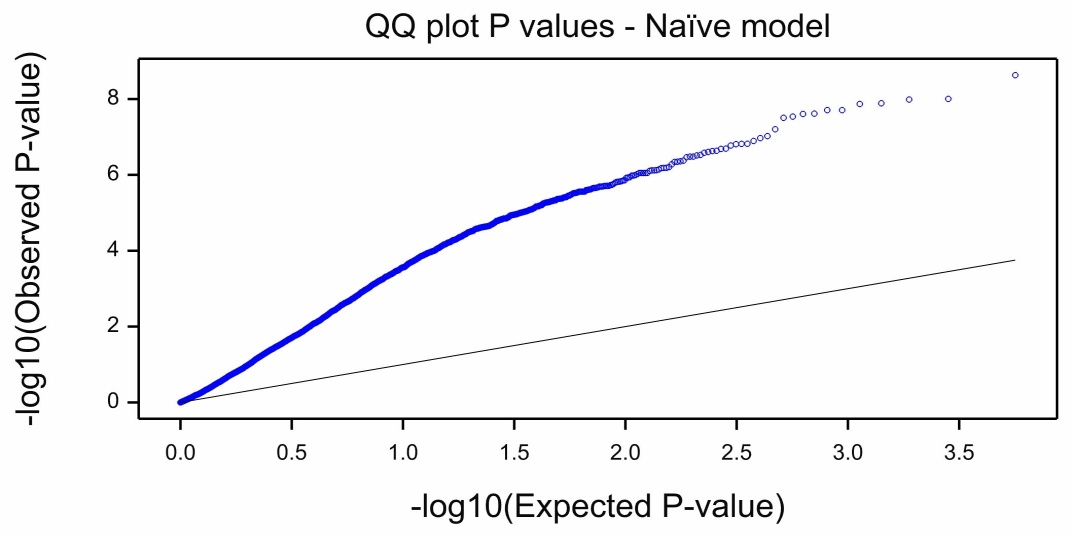


**Figure S1.** Quantile-Quantile (QQ) plots of the observed versus expected *p-*values of the GWAS results related to *U. pisi* inoculation experiments, using a model accounting for population structure (Eigen), a model accounting for familiar relatedness (Kinship), and a naïve model.
