# Supplementary material for: Association Mapping of Lathyrus sativus Disease Response to Uromyces pisi Reveals Novel Loci Underlying Partial Resistance
Source: Front Plant Sci. 2022 Mar 24;13:842545. doi: 10.3389/fpls.2022.842545 (PMC8988034; doi:10.3389/fpls.2022.842545)
Supplement: Supplementary file 2 [file Data_Sheet_2.docx]

Supplementary Material


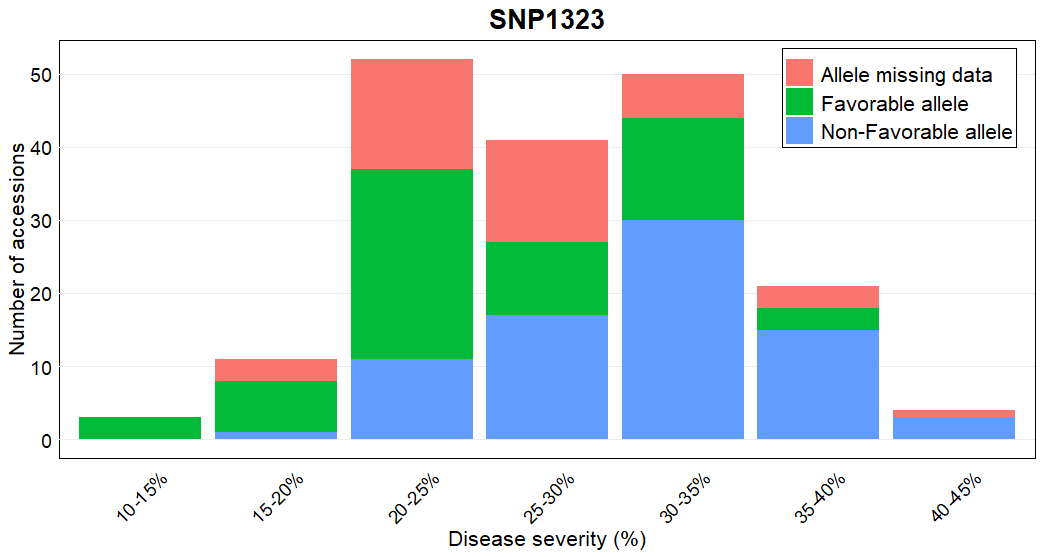

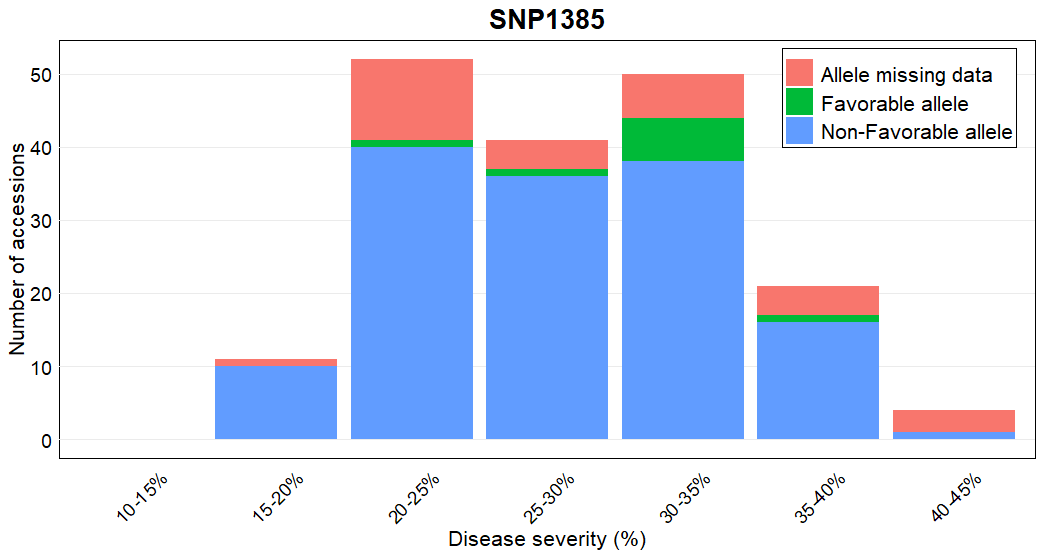

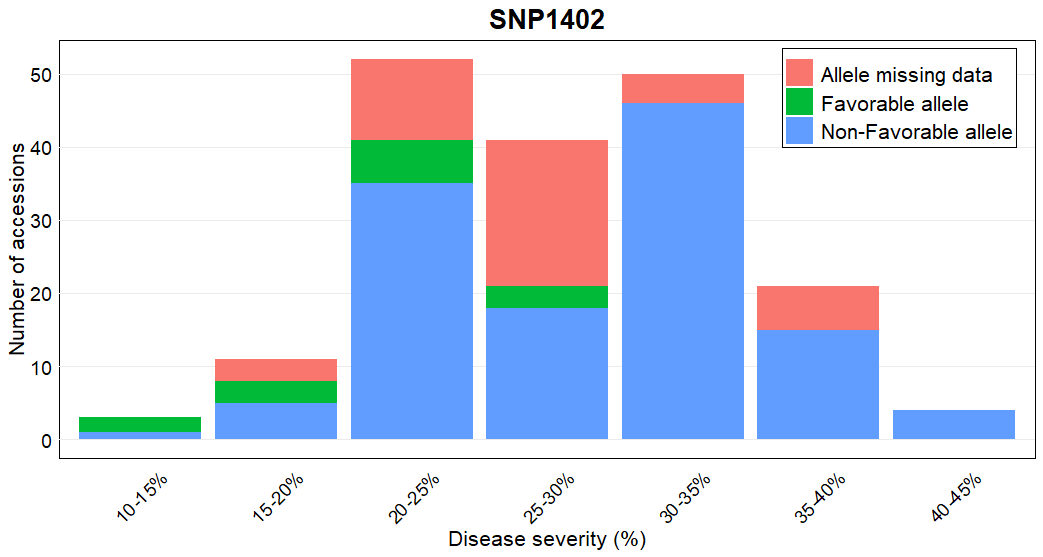

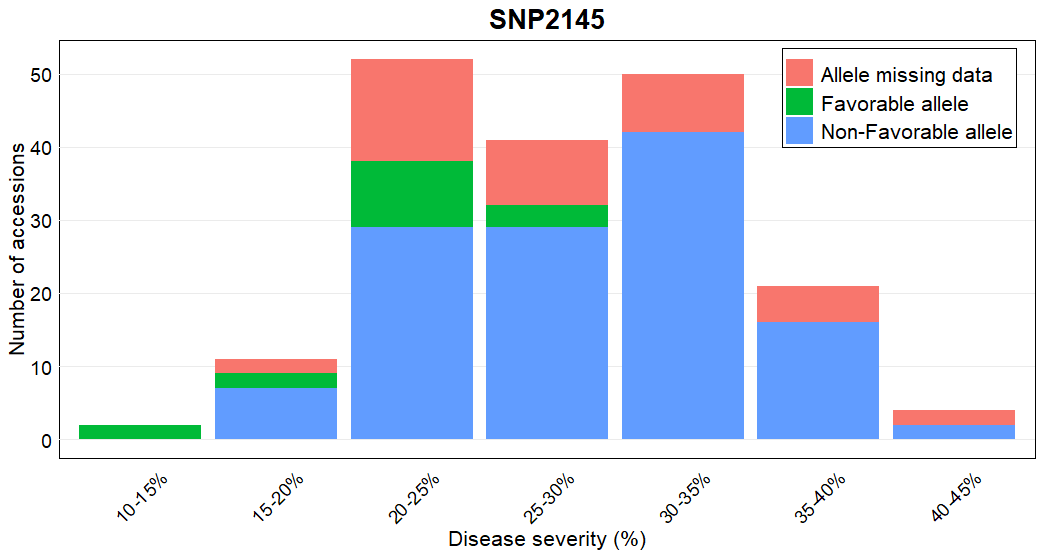

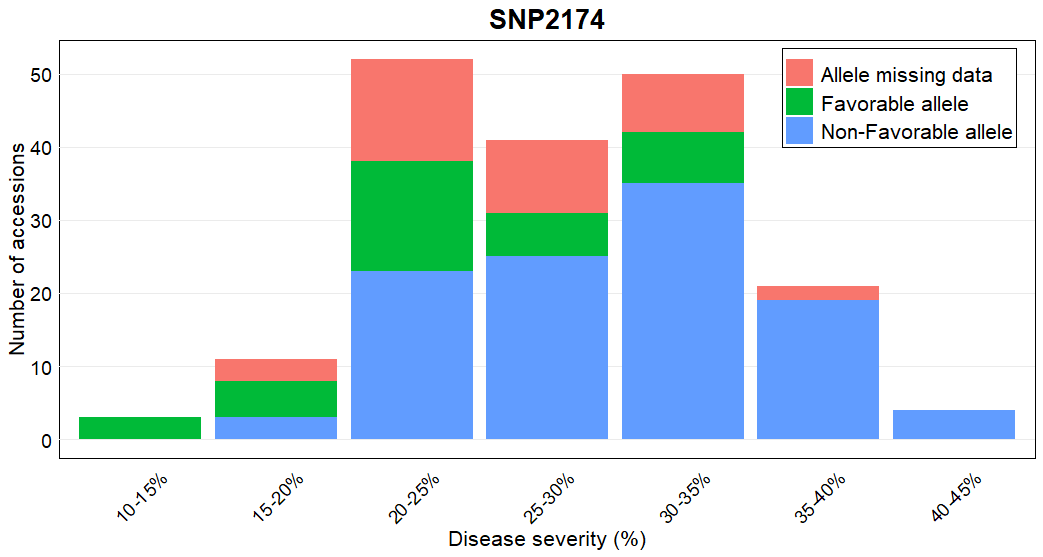

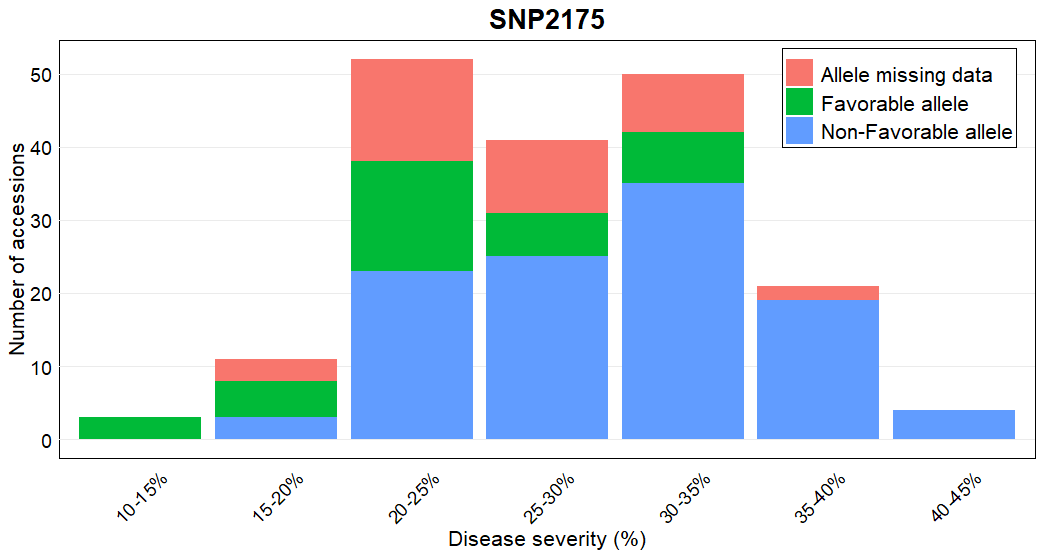

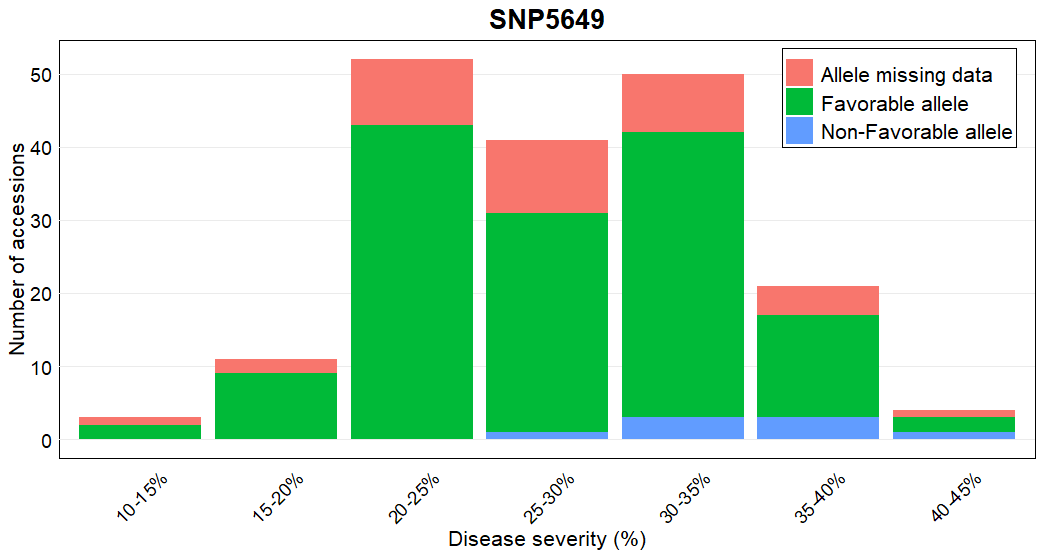


Figure S2. Allele frequency distribution at the DS range scale, of the associated SNPs detected in the GWAS in response to *U. pisi*. Allele missing data refers to the absence of genotypic data.
