# Supplementary material for: Association Mapping of Lathyrus sativus Disease Response to Uromyces pisi Reveals Novel Loci Underlying Partial Resistance
Source: Front Plant Sci. 2022 Mar 24;13:842545. doi: 10.3389/fpls.2022.842545 (PMC8988034; doi:10.3389/fpls.2022.842545)
Supplement: Supplementary file 3 [file Data_Sheet_3.docx]

Supplementary Material

A) Psat6g006240 B) Psat6g010840


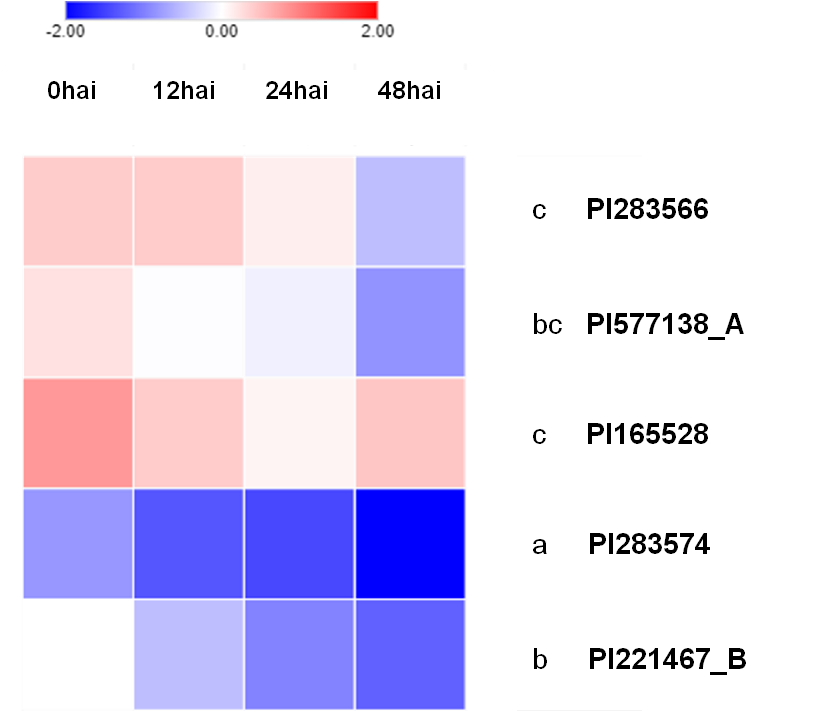

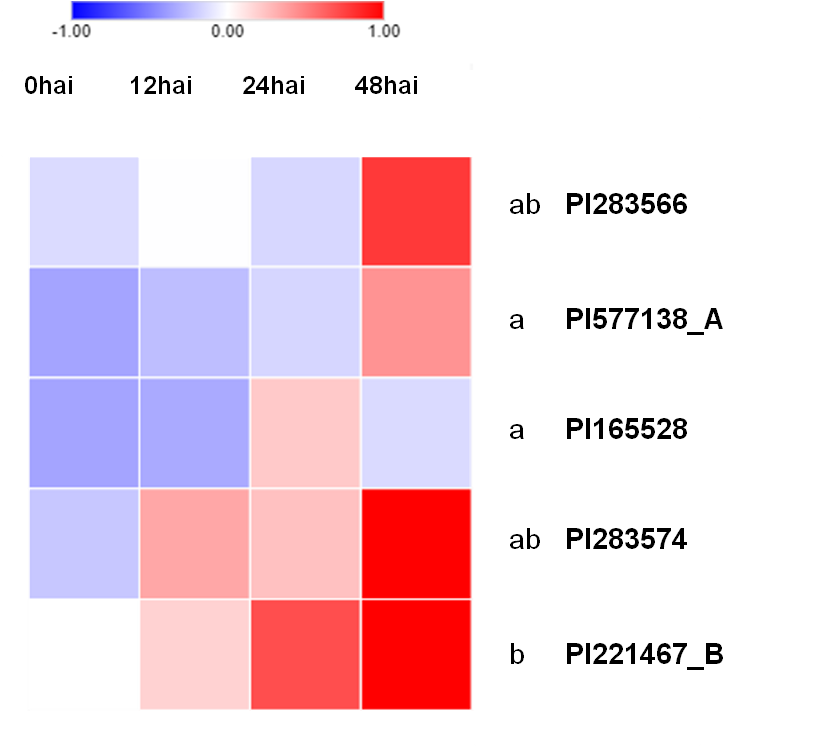


C) Psat4g145320


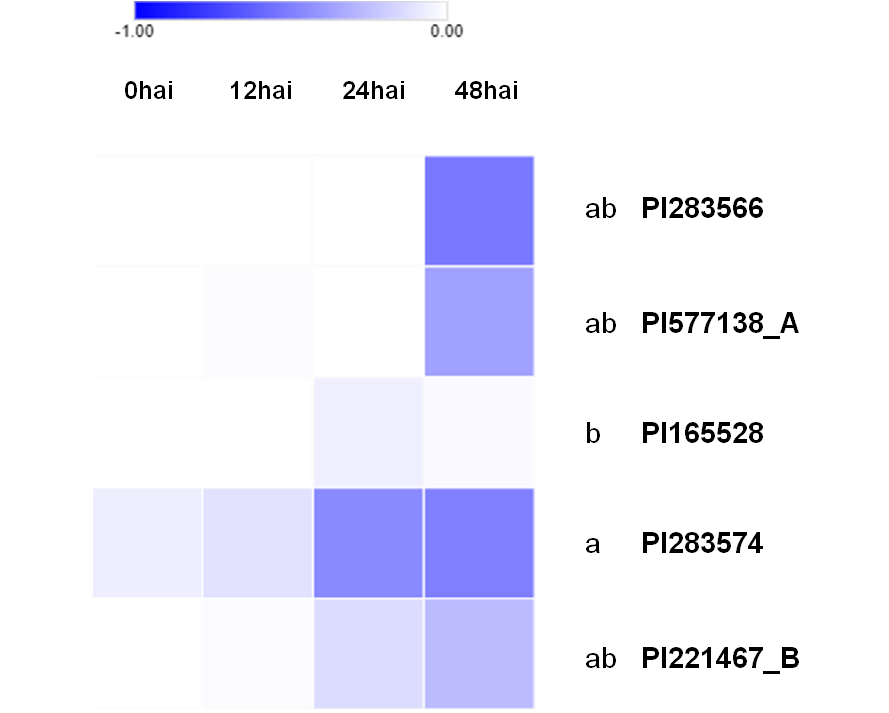


**Figure S3**. Heat map depicting mean log_2_-fold changes of gene expression levels. Relative gene expression analysis of candidate gene related to *U. pisi* disease response (A-C) was analyzed in accessions susceptible (PI283574, and PI221467_B) and partially resistant (PI165528, PI283566, and PI577138_A). Relative expression values were normalized to the mean of the non-inoculated control (0 HAI) of the most susceptible accession (PI221467_B). Higher levels of relative gene expression (log_2_-fold changes > 0) are indicated in red and lower levels (log_2_-fold changes < 0) are indicated in blue. Small letters represent significant differences (*P*-value <0.05), among accessions or time-points (0 HAI, 12 HAI, 24 HAI, 48 HAI).
